# Membranes Based on Cellulose Nanofibers and Activated Carbon for Removal of *Escherichia coli* Bacteria from Water

**DOI:** 10.3390/polym9080335

**Published:** 2017-08-03

**Authors:** Mohammad Hassan, Ragab Abou-Zeid, Enas Hassan, Linn Berglund, Yvonne Aitomäki, Kristiina Oksman

**Affiliations:** 1 Cellulose and Paper Department & Centre of Excellence for Advanced Sciences, National Research Centre, 33 El-Behouth street, Dokki, Giza 12622, Egypt; r_abouzeid@yahoo.com (R.A.-Z.); enasali69@yahoo.com (E.H.); 2 Department of Engineering Sciences and Mathematics, Luleä University of Technology, SE 97187 Luleä, Sweden; linn.berglund@ltu.se (L.B.); yvonne.Aitomaki@ltu.se (Y.A.); kristiina.oksman@ltu.se (K.O.)

**Keywords:** cellulose nanofibers, palm fruit stalks, activated carbon, membrane, water purification, *E. coli*

## Abstract

Cellulosic nanomaterials are potential candidates in different areas, especially in water treatment. In the current work, palm fruit stalks cellulose nanofibers (CNF), TEMPO-oxidized CNF (OCNF), and activated carbon (AC) were used to make thin film membranes for removal of *E. coli* bacteria from water. Two types of layered membranes were produced: a single layer setup of crosslinked CNF and a two-layer setup of AC/OCNF (bottom) and crosslinked CNF (up) on hardened filter paper. The prepared membranes were evaluated regarding their microstructure and layers thickness using scanning electron microscopy (SEM). Water flux and rejection of *E. coli* bacteria was tested using dead end stirred cells at 1 MPa pressure. Thickness of the cosslinked CNF layer in both types of membranes was about 0.75 micron. The results showed that exchanging water by isopropyl alcohol before drying increased porosity of membranes, and thus resulted in increasing pure water flux and flux of bacteria suspension. The two-layer AC/OCNF/CNF membrane had much higher water flux than the single layer CNF due to higher porosity seen on the surface of the former. Both types of membranes showed high capability of removing *E. coli* bacteria (rejection ~96–99%) with slightly higher efficiency for the AC/OCNF/CNF membrane than CNF membrane. AC/OCNF/CNF membrane also showed resistance against growth of *E. coli* and *S. aureus* bacteria on the upper CNF surface while the single layer CNF membrane did not show resistance against growth of the aforementioned bacteria.

## 1. Introduction

Water purification from harmful bacteria using cheap and eco-friendly filters or membranes is of increasing interest. Different types of polymers are used for making membranes that require dissolving polymers in large quantities of solvents then casting the membranes and evaporating the solvents, the so called phase inversion technique. Cellulose nanofibers (CNF) can be isolated from different lignocellulosic materials including wood or agricultural residues [1,2]. These nanofibers can be shaped into porous membranes with tiny pore size without the need to use solvents and casting as in the phase inversion technique. There is an increasing interest recently in using cellulose nanofibers as naturally occurring and renewable material in ultrafiltration membranes, especially their use in making porous ultra-thin membranes (less than one micron) on different kinds of supports [3]. This was motivated by the strength properties of cellulose nanofibers, their amenability for surface modification, and ease of formation of thin membranes with highly porous structure where the pore size is in the sub-micron range [4]. In addition, the use of hydrophilic materials such as cellulose nanofibers reduces the fouling tendency of membranes as many contaminants are hydrophobic in nature and adhere to the hydrophobic surface [5]. 

Durability of thin membranes made from CNF during the ultrafiltration process is important. This requires reasonable thickness of the membranes. As the thickness of the CNF membrane increases the flux of water or water-containing contaminants drastically decreased; this makes the filtration process economically non-feasible. In the literature, membranes from CNF with different thickness and thus different water flux values have been reported; different pressure values for measuring water flux were also used. For example, ultra-thin membranes (100–200 nm) of cellulose nanofibers were prepared by spry drying or doctor blade coating for filtration of dextran [6]. In the case of spray dried membranes, the flux rate ranged from 69 to 230 L/h/m^2^ at a pressure of 69 kPa and the percentage rejection of dextran was 65–75%, while in the case of knife coating, the flux rate ranged from 34 to 145 L/h/m^2^ and percentage rejection of dextran was 62–72%. A CNF thin membrane of about 500–560 nm thickness was formed on a cellulose acetate porous support and used for removal of 10 nm gold and 12 nm ferritin nanoparticles with an efficiency of about 93% [7]. On using 0.8 bar pressure, the water flux ranged from 2750 to 1190 L/h/m^2^/bar. In that study, only 15 mL of solution were used for calculation of the flux rate; this may account for the very high flux rate measured. 2,2,6,6-tetramethyl-1-piperidine oxoammonium (TEMPO)-oxidized CNF or CNF in crosslinked polyethene glycol acrylate (PEGA) layers were formed on polyacrylonitrile/polyethylene terphthalate (PAN/PET) porous matrix [8]. The thickness of CNF layer was about 130 nm while the thickness of CNF/crosslinked PEGA was about 180 nm. At 207 kPa pressure, the water flux of the membranes ranged from 85 to 10 L/h/m^2^; the flux decreased as percentage of the crosslinked polymer increased and the rejection of 0.1% PEG 4600 ranged from about 50 to 98% depending on the concentration of the crosslinked PEGA. TEMPO-oxidized CNF ultra-thin membrane with a thickness of 0.5 µm was formed over PAN/PET support and used for filtration of microsphere latex suspension (0.1 µm diameter), sodium alginate polymer solution, and oil-in-water emulsion [9]. At a pressure of 207 kPa, the flux was dependent on the particles to be separated and was about 303, 208, and 199 L/h/m^2^ in the case of latex suspension, oil-in-water emulsion, and sodium alginate solution, respectively. Polyamide-amine-epichlorohydrin(PAE)-crosslinked cellulose nanofibers thin membranes of about 15–47 µm thickness were formed on a paper sheet. The membranes were prepared in presence or absence of silica nanoparticles and tested for filtration of polyethylene glycol (PEG) [10]. At 200 kPa, water flux of membranes without silica was in the range of about 20–100 L/h/m^2^ while optimized membranes containing silica showed water flux from 80 to 110 L/h/m^2^. Rejection of PEG 200–300 KDa was above 95% and was similar to the membranes made without silica nanoparticles. Nanopaper sheets from CNF and TEMPO-oxidized CNF with basis weight from 20 to 80 g/cm^2^ were prepared and tested for filtration of PEG having molecular weight of 1 to 95 KDa [11]. Water flux values for nanopaper membranes with high grammage (80 gsm) was very low (1–5 L/m^2^/h/MPa), while for membranes with low grammage (20 g/m^2^), i.e., lower thickness, water flux ranged from 4–20 L/h/m^2^/MPa. High rejection values (>90%) were achieved for PEG with molecular weight >10 KDa.

Drying conditions of cellulose nanofibers membranes play important role in determining their porosity, and thus water flux of these membranes, since removal of water during drying of membranes resulted in formation of a tight and narrow pore size structure leading to considerably low flux. Changing the porosity of membranes can be a tool for adapting them for specific applications. In the previous published work, there is no specific or unified protocol used for drying CNF thin membranes. In some studies, the wet CNF membranes were dried by heating and in other cases water in the membranes was exchanged by other less polar solvents, then oven dried. Few studies investigated the effect of solvent exchange of water in the wet membranes on water flux and removal of contaminates. For example, Karim et al. prepared two-layer membranes from CNF (~176 µm thick) and functionalized cellulose nanocrystals (25 µm thick); the wet membranes were hot pressed or exchanged with acetone before hot pressing. Membranes prepared by exchanging water with acetone gave higher water flux values due to the higher surface area and porosity of these membranes. At 450 kPa, water flux ranged from 6 to 25 L/h/m^2^/MPa; the membrane was used for removal of silver, copper, and iron metal ions [12]. In other studies, non-crosslinked and calcium chloride- or trisodium trimetaphosphate crosslinked 2,3-dicarboxylic acid CNF thin membranes with a thickness of about 0.85 µ were formed over a PAN/PET scaffold and used for filtration of dextran [13,14]. The wet membranes were exchanged with alcohol before drying. For the 0.85 µm non-crosslinked membrane under pressure of 1 to 3 bar, pure water flux ranged from 30 to 140 L/h/m^2^/bar and the percentage rejection for 35–45 KDa dextrane was 74–85%. In the case of 0.85 µm calcium chloride and sodium tripolyphosphate crosslinked membrane under pressure of 1 to 3 bar, pure water flux ranged from 70–125 L/h/m^2^/bar to 170–225 L/h/m^2^/bar, respectively, and rejection of dextran ranged from 20–90% to 10–50%, respectively.

Although pore diameter of membranes made from CNF is known to be less than that of bacteria, the evaluation of the filtration process (flux rate and rejection) of bacteria in aqueous solutions using cellulose nanofibers was rarely studied. For the best of our knowledge, the only published work was preparation of functionalized cellulose nanofibers membranes on PAN/PET support for removal of viruses and bacteria [15]. The CNF were functionalized through grafting by amines, such as polyvinyl amine, polyethylene imine, or ethyl amine. A volume of 10 mL of *E. coli* suspension (106 CFU/mL) was used to evaluate the efficiency of membrane to remove bacteria at a constant flow rate of 192 L/h/m^2^ and the results showed complete removal of bacteria. The effect of presence of bacteria in water on water flux was not studied in that work.

Activated carbon (AC) is a highly porous material with high surface area; it is widely used in water treatment to remove different pollutants such as dyes, heavy metals, and many organic contaminants [16]. Previous studies showed the ability of AC to remove *E. coli* bacteria from tap water by adhesion [17,18]. Modifications of activated carbon with several nanoparticles such as sliver, titanium dioxide, and many others have been reported as antibacterial materials for water treatment [19,20,21]. 

The aim of the current work is to use CNF, TEMPO-oxidized CNF isolated from palm fruit stalks, and activated carbon in making structured thin film membranes for water purification of *E. coli* bacteria. AC was used as the highly porous bottom layer material to induce formation of more pores in the top layer of CNF to increase water flux of the membrane. TEMPO-oxidized CNF was used in the bottom layer with AC to improve dispersion and distribution of AC in the membrane formed and also to improve its antibacterial resistance. The effect of exchanging water with alcohol before drying of wet membranes on water flux and rejection was also studied.

## 2. Materials and Methods 

### 2.1. Materials

Date palm fruit stalks were collected from local fields in Giza, Egypt after separating dates from the stalks. 2,2,6,6-tetramethyl-1-piperidine oxoammonium salt (TEMPO), sodium hypochlorite, sodium hydroxide, sodium chlorite, acetic acid, ethanol, isopropyl alcohol, and sodium bromide were reagent grade chemicals and used as received (Sigma-Aldrich, St. Louis, MO, USA). Polyamide-amine-epichlorohydrin (PAE) was commercial grade (~33%, *w*/*w* solid content, Solines, Wilmington, DE, USA). PAE solutions were diluted to 1 wt % with distilled water prior to the experiment. 

### 2.2. Preparation of Palm Fruit Stalks Pulp

Bleached pulp was prepared from the stalks as described before [22]. Date palm fruit stalks pulp was prepared by alkali treatment using 15% NaOH (*w*/*w*, based on oven-dried stalks) at 150 °C for 2 h. The produced pulp was bleached using sodium chlorite/acetic acid mixture at 80 °C for 1 h [23]. Chemical composition of the bleached pulp was determined according to the previously published methods [24] and was: α-cellulose 71.5%; pentosans 18.4%; degree of polymerization (DP) 1264; and 0.64% ash.

### 2.3. Isolation of Cellulose Nanofibers (CNF) 

Isolation of CNF from bleached pulp was carried out similar to the previously published protocol [25]. In brief, bleached pulp was first disintegrated by a high-shear mixer using pulp suspensions of 2% consistency. The fibers were then refined using a high-shear ultrafine friction grinder, or a so-called supermasscolloider (MKCA6-2, Masuko Sanguo, Kawaguchi, Japan); the gap between the disks was adjusted to 9 µm and the fibers were run through the grinder for about 100 min.

### 2.4. Isolation of TEMPO-Oxidized CNF

The method used for oxidation of pulp fibers was based on that of Saito et al. [26]. Bleached pulp (3 g) was dispersed in distilled water (400 mL) with TEMPO (0.048 g, 0.3 mmol) and sodium bromide (0.48 g, 4.8 mmol). Then 30 mL of sodium hypochlorite solution was then added with stirring and the pH was adjusted to 10. At the end of reaction, the pH was adjusted to 7 and the product was centrifuged at 10,000 rpm. The product was further purified by repeatedly adding water, dispersion, and centrifugation. Finally the product was purified by dialysis for one week against de-ionized water with 3500 MWCO Spectra/Por dialysis tubing. TEMPO-oxidized fibers were homogenized using a two-chamber high-pressure homogenizer (APV-2000, SPX, Silkeborg, Denmark) after being diluted with water to 1% consistency and passed twice through the device. The pressure was kept at 40 bar in one chamber and 400 bar in the other chamber. Carboxylic content of the oxidized nanofibers was 0.42 mmol/g according to the Technical Association of the Pulp and Paper Industry (TAPPI ) test method T237cm-98.

### 2.5. Characterization of CNF and TEMPO-Oxidized CNF

Transmission electron microscopy was carried out using a high-resolution transmission electron microscope (JEM-2100 transmission electron microscope, JEOL, Tokyo, Japan). Microscopic features of membranes were investigated using a FEI Quanta 200 scanning electron microscope (FEI Company, Eindhoven, The Netherlands) at an acceleration voltage of 20 kV.

### 2.6. Preparation and Characterization of Activated Carbon

Activated carbon was prepared from olive stones by heat treatment of ground olive stones/KOH mixture at 275 °C for 45 min followed by heat treatment of the same mixture at 600 °C for 90 min under nitrogen atmosphere [27]. An olive stones/KOH weight ratio of 1:1 was used. The obtained activated carbon was washed well with distilled water to remove excess alkali, filtered and dried in an oven at 105 °C for 4 h. Nitrogen adsorption experiments were conducted to determine the specific surface area using Quantachrome Nova-1200 instrument (Quantachrome Instruments, Boynton Beach, FL, USA). The sample was out gassed overnight at 180 °C prior to adsorption measurements. The BET model was applied to fit the nitrogen adsorption isotherms and evaluate the specific surface area of the sample. Microscopic features were investigated using high-resolution scanning electron microscope (Zeiss Merlin FEG-SEM, Zeiss, Oberkochen, Germany). Zeta potential was measured using Zetasizer Nano-ZS90 (Malvern Instruments, Malvern Worcestershire, UK). 

### 2.7. Nanocellulose Membranes

**Scheme 1:**
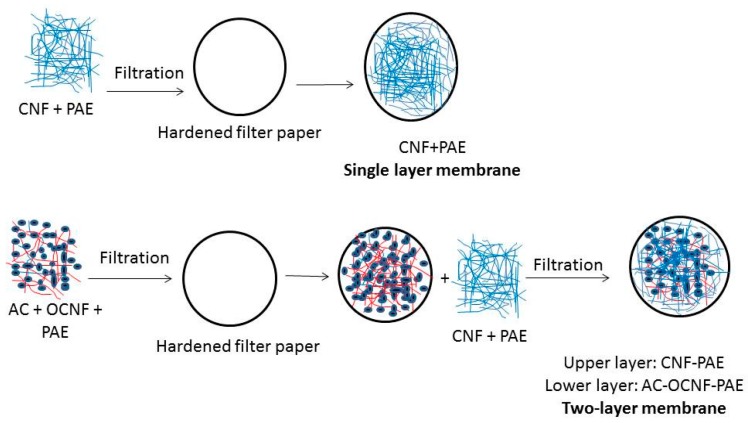
Preparation of single layer cellulose nanofibers (CNF) and two-layer CNF/AC (activated carbon) membranes on hardened filter paper.

Two types of membranes were prepared on hardened filter: a single layer crosslinked CNF (named as CNF membrane) and a two-layer membrane where the bottom layer is an AC-rich layer and the top layer is crosslinked CNF (named as CNF/AC membrane, Scheme 1).

In the case of the single-layer membrane, 0.02 g CNF in water suspension with a concentration of 0.1 wt % and 4% PAE crosslinker (based on oven-dry weight of CNF) were filtered on 9-cm hardened filter paper using a vacuum pump. The prepared membrane was either dried directly in an oven with circulating hot air at 105 °C for 30 min, or water in the wet membrane was first exchanged by isopropyl alcohol then dried at 105 °C for 30 min. The grammage of the formed CNF membrane was 2.5 g/m^2^. 

In the case of the two-layer membrane, the bottom layer was prepared by mixing 0.03 g of AC, 2 wt % (based on wt of AC) TEMPO-oxidized CNF suspension with a concentration of 0.1 wt %, 1% PAE crosslinker (based on wt of CNF); the mixture was sonicated for two minutes using an ultrasonic processor (hielscher UP400s, Teltow, Germany) and then filtered on the hardened filter paper. The wet formed layer is either directly dried in oven at 105 °C for 30 min or exchanged with alcohol then oven dried at 105 °C for 30 min. To form the top layer, 0.02 g CNF in water suspension with a concentration of 0.1 wt % and 4% PAE crosslinker (based on oven-dry weight of CNF) were filtered on the hardened filter paper with the AC-rich layer. The wet formed layer is either directly dried in oven at 105 °C for 30 min or exchanged with isopropyl alcohol then oven dried at 105 °C for 30 min.

### 2.8. Test Microorganism Strain

*Escherichia coli* was obtained from a culture collection and grown on the tryptic soy agar medium and incubated at 37 °C for 48 h, the prepared slants were stored at 4 °C until required. A loop full of bacteria from agar slant was taken and inoculated into 50 mL of tryptic soy broth medium in 125 mL flask then the flask was incubated at 120 rpm in an incubator shaker at 37 °C for 24 h. Two different dilutions of *E. coli* were prepared by addition of 2 mL of the previous culture to 250 and 500 mL of sterile distilled water. The optical density (OD) of the prepared suspensions measured by UV-visible spectrometer at 600 nm was about 0.12. 

### 2.9. Evaluation of Membranes Properties

#### 2.9.1. Pure Water Flux

The water flux of the membranes was measured using a dead end stirred cell, (Sterlitech HP 4750, Sterlitech, Kent, WA, USA). Prior to the measurements, discs with a diameter of about 5 cm were cut out from the membranes and soaked in water for one hour to ensure equilibration of the membrane. The conditioned membranes were placed in the dead end cell on a stainless steel porous support disk and water was passed through the membranes at room temperature at a differential pressure of 1 MPa maintained using N_2_ gas. The quantity of water that passed through the membrane for a defined time interval was weighed accurately and the flux was calculated (L/h/m^2^) for the active filtration area (14.6 cm^2^).

#### 2.9.2. Rejection Efficiency

The capability of bacteria removal by the prepared membranes was evaluated using *E. coli* water suspension. Optical density at 600 nm (OD_600_) of the bacteria suspension was about 0.12. The suspension was filtered in the dead end cell as in the case of measuring water flux mentioned above. The filtrate was collected and the turbidity of the cell suspension was examined using UV-visible spectrometer (Shimadzu, Tokyo, Japan) and expressed as OD. The capability of the prepared membranes to remove *E. coli* from water was calculated using the following formula:

Rejection (%) = [(Control OD_600_ − sample OD_600_)/Control OD_600_] × 100%.

#### 2.9.3. Antibacterial Resistance 

The antibacterial activity of the membranes was investigated against Gram-negative bacteria *Escherichia coli* and Gram-positive bacteria *Staphylococcus aureus* by the disk diffusion method. The membranes were cut into square shapes with 10 mm sidelength and sterilized by ultraviolet lamp for 60 min. Then 1.0 mL of each inoculum containing 10^8^–10^9^ cfu/mL was spread on the tryptic soy agar plate with a sterile cotton swab and the membrane samples were applied to the agar plates. The plates were incubated at 37 °C for 24 h before examination of the inhibition zone. 

## 3. Results and Discussion

### 3.1. Cellulose Nanofibers 

**Figure 1:**
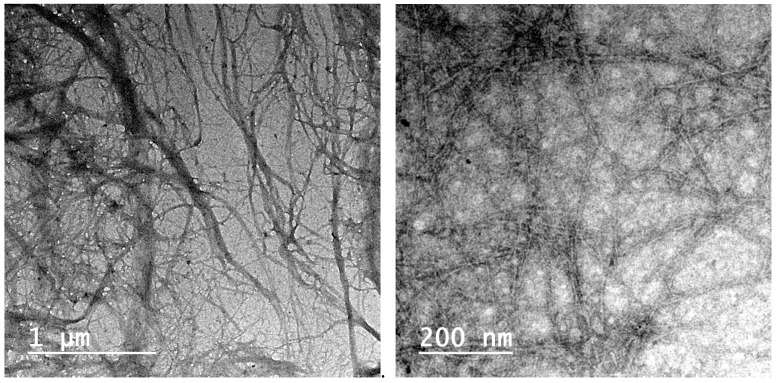
TEM of CNF (**left**) and TEMPO-oxidized CNF (**right**) isolated from date palm fruit stalks pulp.

CNF isolated from palm fruit stalks using ultrafine grinding showed width in the range of about 13–25 nm; fibrils with larger width of about 75–100 nm were also noticed. On the other hand, TEMPO-oxidized nanofibers prepared by oxidation of bleached palm pulp by TEMPO/NaOCl followed by high pressure homogenization showed a thinner width of about 3–8 nm (Figure 1). 

### 3.2. Activated Carbon Prepared from Olive Stones

**Figure 2:**
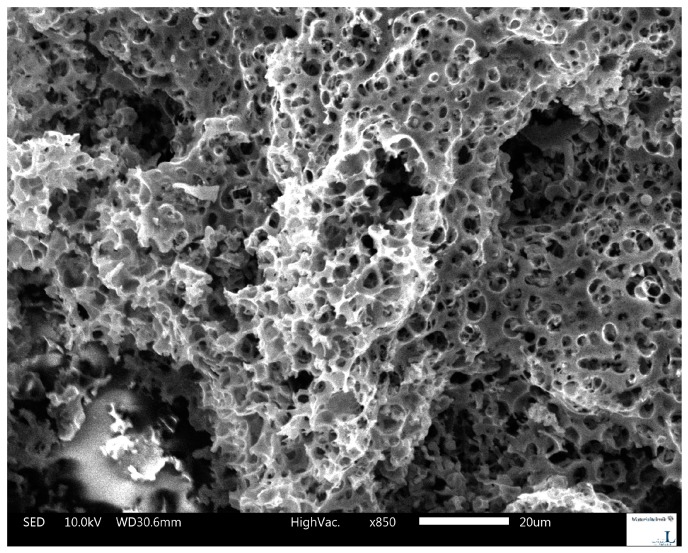
SEM image of activated carbon prepared from olive stones. The scale bar stands for 20 µm.

Activated carbon (AC) used in the current work was prepared from olive stones and showed a highly porous structure. As determined by BET nitrogen adsorption, the surface area of the prepared AC was 464 g/m^2^, a total pore volume was 0.3 cm^3^/g, and the average pore diameter was 2.41 nm. The large cavities shown in SEM image (Figure 2) had a narrow range diameter of 2–4 µm. Zeta-potential measurement showed a negatively charged surface with zeta potential value of −11.5 ± 6.0 mV.

### 3.3. CNF and CNF/AC Membranes

Two types of membranes were formed over a hardened filter paper: (1) single layer of crosslinked CNF membrane and (2) two-layer membrane consisting of a bottom layer of AC-rich TEMPO-oxidized CNF/AC layer and a top layer of crosslinked CNF. The basis weight of the CNF top layer was about 2.5 g/m^2^. The role of TEMPO-oxidized CNF in the bottom layer was to disperse AC to form an evenly distributed layer on the filter paper and to impart its antibacterial resistance. The reason for using the porous AC in the bottom layer was mainly to induce formation of a more porous CNF membrane in the upper layer. AC with its high porosity and surface area can also adsorb water contaminants and bacteria.

In addition, since drying conditions significantly affect porosity of CNF membranes, they were either directly oven-dried at 105 °C for 30 min under air circulation or washed with isopropyl alcohol to exchange water then oven dried at 105 °C for 30 min.

**Figure 3:**
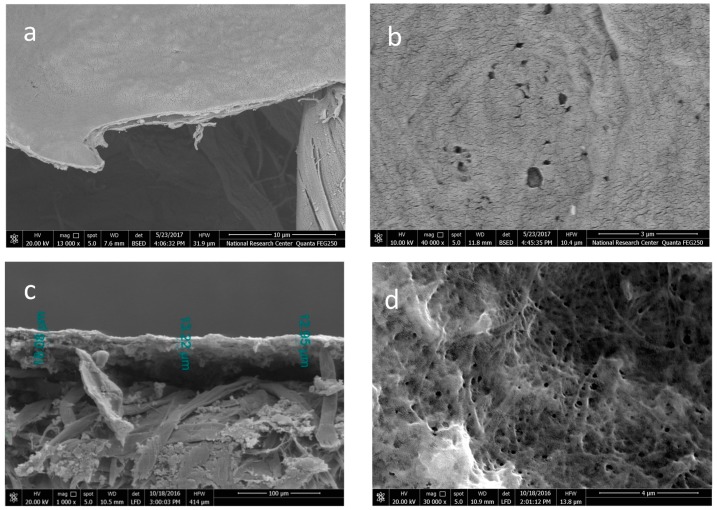
The cross section and surface of the oven-dried CNF membrane (**a**,**b**) and the CNF/AC membrane (**c**,**d**) formed on hardened filter paper.

Figure 3 shows SEM images of the CNF and CNF/AC membranes prepared by direct oven drying. The images showed that the thickness of the CNF layer in the single layer membrane was about 0.75 µm while in case of the CNF/AC membrane the thickness of the two layers (AC and CNF) formed was about 13 µm; the major thickness of the AC layer was due to the presence of AC particles since the same weight of CNF was used in both types of membrane. SEM images also showed that the CNF/AC membrane had higher porosity at the surface than the single layer CNF membrane. In fact, no porous structure was obtained in the case of the oven-dried CNF membrane and mainly holes appeared at the surface; the diameter of these holes ranged from about 115 to 315 nm. On the other hand, the diameter of the pores at the surface of CNF/AC membrane was in the range from about 145 to 251 nm. 

**Figure 4:**
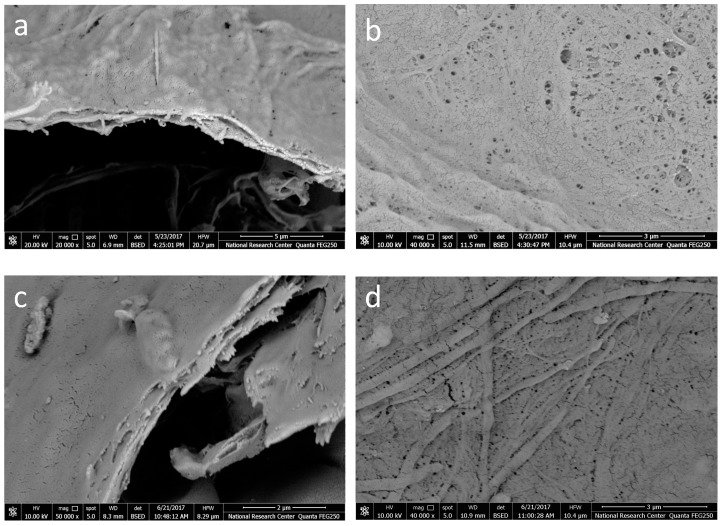
SEM images of cross section and surface of the alcohol-exchanged CNF membranes (**a**,**b**) and the CNF/AC membranes (**c**,**d**) formed on hardened filter paper.

Figure 4 shows SEM images of the alcohol-exchanged CNF and CNF/AC membranes formed on hardened filter paper. The images showed that alcohol-exchanged CNF membranes had much higher porosity at the surface than the oven-dried ones. This could be due to much more hydrogen bonding generated between the nanofibers in the case of direct oven drying than in case of the alcohol-exchanged membrane. The diameter of the pores at the surface of alcohol-exchanged CNF membrane ranged from about 61 to 172 nm. In the case of CNF/AC membrane, the pores at the surface of the alcohol-exchanged membrane appeared narrower than those seen in case of the oven-dried membrane (from 50 to 100 nm).

### 3.4. Water Flux of Membranes

**Figure 5:**
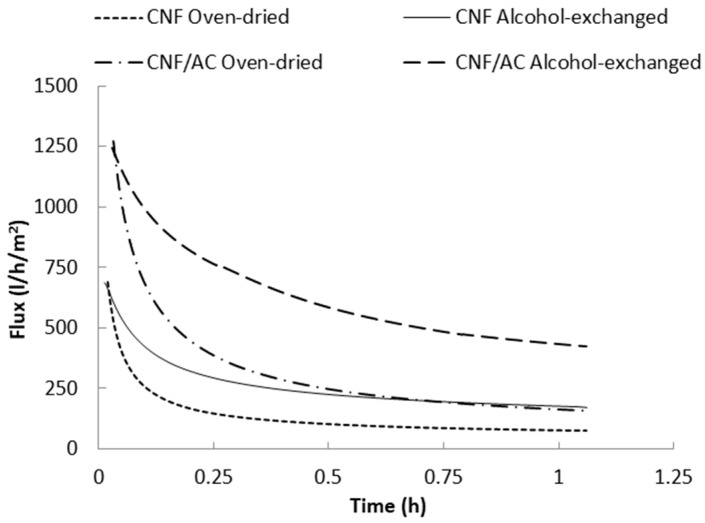
Water flux of the CNF and CNF/AC membranes dried with and without isopropyl alcohol exchange.

Figure 5 shows water flux versus time curves of the single layer CNF and the two-layer CNF/AC membranes. As shown in the figure, water flux of the different membranes significantly decreased at the beginning of the measurement due to compaction of the CNF by the action of the applied pressure, and thus porosity decreased [28,29]. Water flux of the oven-dried two layer CNF/AC membrane was much higher than the oven-dried CNF membrane. This could be due to the higher porosity of the CNF/AC membrane than that of the CNF one, as shown in the SEM images above. After one hour of filtration, the average water flux was 55 and 158 L/h/m^2^ in the case of the CNF and CNF/AC membranes, respectively. It should be noted that the flux values mentioned are at the time studied and not the stable flux values since some curves still show slopes at end of the test.

Exchanging water with alcohol before drying significantly affected water flux of the formed membranes. After one hour of filtration, the average water flux of the alcohol-exchanged CNF and the CNF/AC membranes were 165 and 425 L/h/m^2^, respectively. This indicates 200% and 169% increasing water flux as a result of alcohol exchange in the case of the CNF and CNF/AC membranes, respectively.

**Figure 6:**
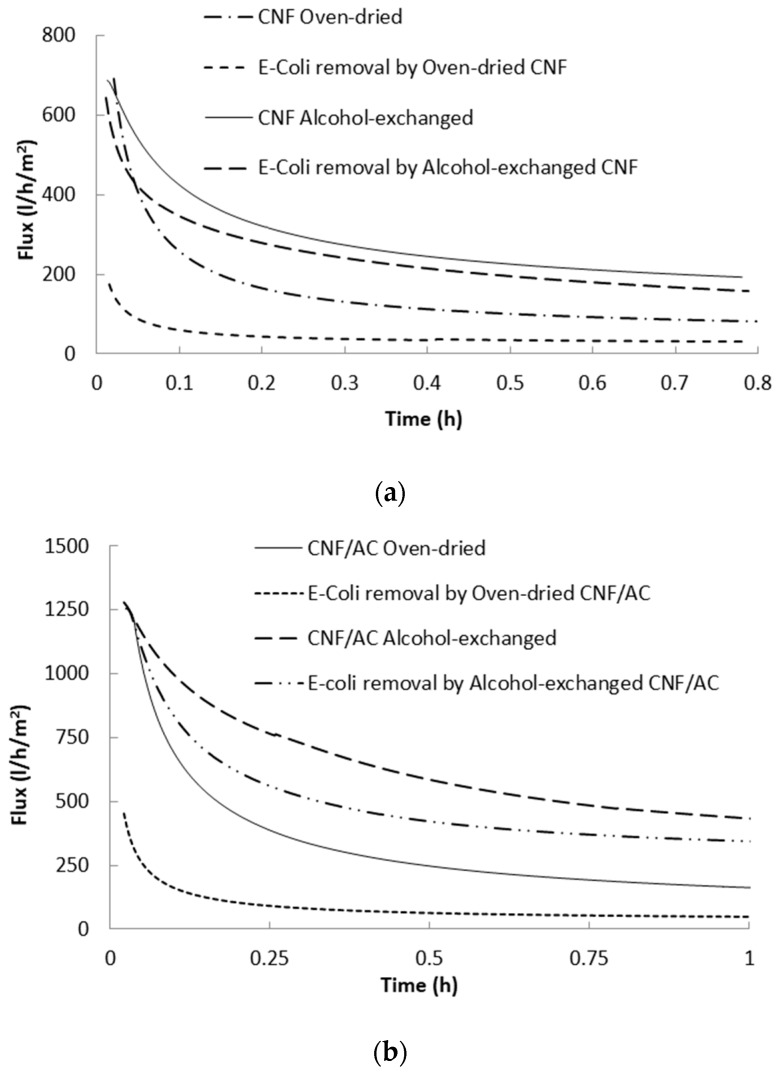
Flux of pure water and *E. coli* bacteria suspension by CNF (**a**) and CNF/AC (**b**) membranes.

The prepared membranes were evaluated for removal *E. coli* bacteria from water. A bacterial water suspension with optical density of about 0.12 was used in the study. The concentration of the bacteria used in the current work was much higher than that usually exists in polluted water. The high concentration was used for easy detection in water. Figure 6 shows the flux of the water suspension containing *E. coli* bacteria. As shown in the figures, the presence of bacteria particles caused reduction of the water flux due to clogging of the pores. In the case of alcohol-exchanged CNF membranes, water flux of *E. coli* suspension was about 139 L/h/m^2^ compared to 165 L/h/m^2^ in the case of pure water (19% decrease in flux), while in the case of the oven-dried CNF membrane water flux of *E. coli* suspension was about 39.3 L/h/m^2^ compared to 55 L/m^2^/h in the case of pure water (29% decrease in flux). The CNF/AC membranes showed higher flux than CNF membranes in filtering bacteria suspension. After about one hour of filtration, flux of bacteria suspension through the alcohol-exchanged CNF/AC membrane was about 345 L/h/m^2^ compared to 425 L/h/m^2^ in the case of pure water (23% decrease in flux), while in the case of the oven-dried CNF/AC membrane water flux of *E. coli* suspension was about 50 L/h/m^2^ compared to 158 L/h/m^2^ in the case of pure water (68% decrease in flux).

### 3.5. Removal Efficiency of E. coli Bacteria 

**Figure 7:**
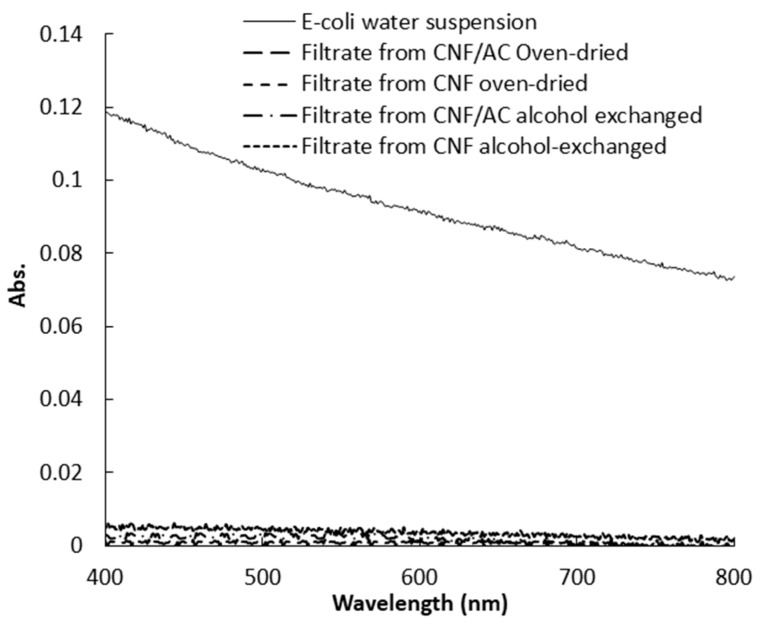
Visible spectra of *E. coli* suspensions before and after filtration through CNF and CNF/AC membranes.

**Figure 8:**
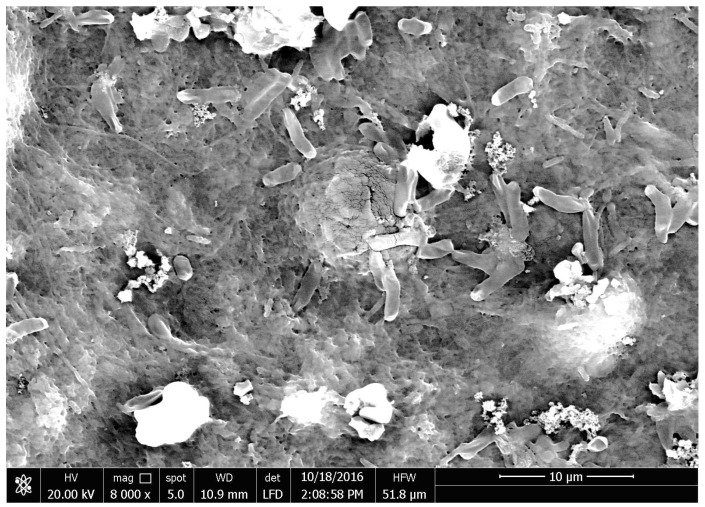
SEM image of oven-dried CNF/AC membrane after filtration of *E. coli* bacteria suspension.

The efficiency of CNF and CNF/AC membranes to remove *E. coli* bacteria was estimated by measuring optical density of a water suspension containing bacteria before and after filtration. Figure 7 shows UV-visible spectra of the bacteria suspension before and after filtration through the CNF and CNF/AC membranes. As shown from the curves, both the CNF and CNF/AC membranes, either oven-dried or alcohol-exchanged, could remove *E. coli* bacteria from water but CNF/AC membranes were slightly more effective than CNF ones (~99% and 97% decrease in absorbance at 600 nm in case of CNF/AC and CNF, respectively). This is due to that the pore size of the different membranes is much smaller than the dimension of the rod shape *E. coli* bacteria. Figure 8 shows SEM image of oven-dried CNF/AC membrane after filtration of the bacterial suspension where *E. coli* bacteria is about 0.7 µm in width and 3.5 µm in length. The slightly higher efficiency of the CNF/AC layer could be due to the presence of AC and TEMPO-oxidized CNF in the bottom layer which can adsorb any organic traces in water used in the test or traces of bacteria that could pass through any imperfection in the upper CNF layer. 

### 3.6. Bacterial Resistance 

**Figure 9:**
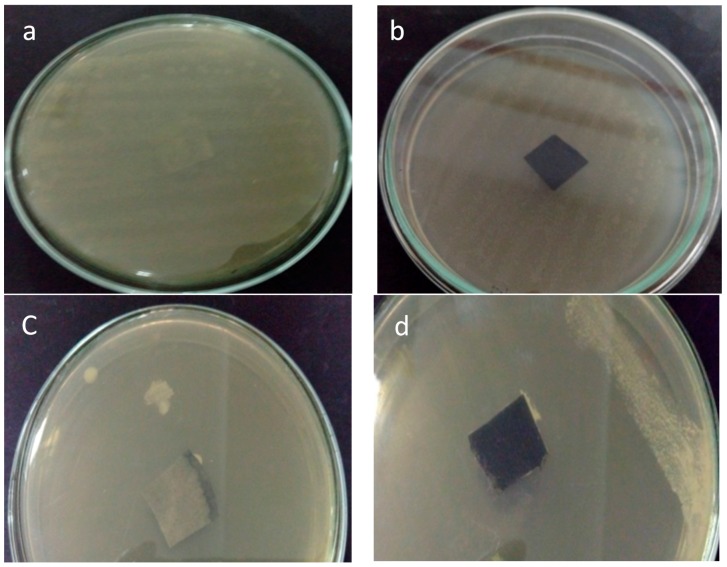
Photos of surfaces of hardened filter paper (**a**,**c**) and CNF/AC membrane (**b**,**d**) subjected to *S. aureus* and *E. coli* inoculums, respectively.

Bacterial resistance is an important property for membranes used for removal of bacteria to prevent membrane deterioration by the action of filtered bacteria and also to prevent growth of bacteria on the surface of membranes. In the current study, bacterial resistance of the CNF/AC membrane was studied using *E. coli* and *S. aureus* bacteria. As shown in Figure 9, after incubation for 24 h at 37 °C, there was no growth of bacteria on the upper CNF layer of the membrane while bacteria could grow on the surface of hardened filter paper (the filter paper surface is totally covered by bacteria). The reason for the antibacterial resistance of the membrane could be due to the presence of TEMPO-oxidized CNF that is used to distribute AC in the bottom layer. Some previous studies stated the bacterial resistance properties of C-6 oxidized cellulose against *E. coli* and other bacteria [30,31,32]. In addition, a previous study on membranes prepared from TEMPO-oxidized CNF proved bacterial resistance of the membrane towards bacteria [9].

## 4. Conclusions

CNF, TEMPO-oxidized CNF, and activated carbon could be used to make structured membranes for efficient removal of *E. coli* bacteria from water. Exchanging water from wet membranes by isopropyl alcohol before drying played an important role in generating more porous structure, and thus more water flux. The use of activated carbon as a bottom layer significantly improve porosity of the upper CNF layer of the two-layer structured membranes, and thus improved water flux. Presence of TEMPO-oxidized CNF in the two-layer structured membrane could improve resistance of the CNF membrane toward growth of bacteria on its surface.

## References

[1] Zhao Y., Moser C., Lindström M.E., Henriksson G., Li J. (2017). Cellulose nanofibers from softwood, hardwood, and tunicate: Preparation-structure-membrane performance interrelation. ACS Appl. Mater. Interfaces.

[2] Hassan M.L., Pandey J.K., Takagi H., Nakagaito A.N., Kim H.-J. (2015). Bagasse and rice straw nanocellulosic materials and their applications. Handbook of Polymer Nanocomposites. Processing, Performance and Application. Volume C: Polymer Nanocomposites of Cellulose Nanoparticles.

[3] Voisin H., Bergström L., Liu P., Mathew A.P. (2017). Nanocellulose-based materials for water purification. Nanomaterials.

[4] Ma H., Burger C., Hsiao B.S., Chu B. (2011). Ultrafine polysaccharide nanofibrous membranes for water purification. Biomacromolecules.

[5] Rana D., Matsuura T. (2010). Surface modification for antifouling membranes. Chem. Rev..

[6] Wang Z., Ma H., Chu B., Hsiao B.S. (2017). Fabrication of cellulose nanofiber-based ultrafiltration membranes by spray coating approach. J. Appl. Polym. Sci..

[7] Soyekwo F., Zhang Q.G., Lin X.C., Wu X.M., Zhu A.M., Liu Q.L. (2016). Facile preparation and separation performances of cellulose nanofibrous membranes. J. Appl. Polym. Sci..

[8] Wang Z., Ma H., Hsiao B.S., Chu B. (2014). Nanofibrous ultrafiltration membranes containing cross-linked poly(ethylene glycol) and cellulose nanofiber composite barrier layer. Polymer.

[9] Ma H., Burger C., Hsiao B.S., Chu B. (2014). Fabrication and characterization of cellulose nanofiber based thin-membrane nanofibrous composite membranes. J. Membr. Sci..

[10] Varanasi S., Low Z.-X., Batchelor W. (2015). Cellulose nanofibre composite membranes—Biodegradable and recyclable UF membranes. J. Chem. Eng..

[11] Mautner A., Lee K.-Y., Lahtinen P., Hakalahti M., Tammelin T., Li K., Bismarck A. (2014). Nanopapers for organic solvent nanofiltration. Chem. Commun..

[12] Karim Z., Claudpierre S., Grahn M., Oksman K., Mathew A.P. (2016). Nanocellulose based functional membranes for water cleaning: Tailoring of mechanical properties, porosity and metal ion capture. J. Membr. Sci..

[13] Visanko M., Liimatainen H., Sirviö J.A., Haapala A.b., Sliz R., Niinimäki J., Hormi O. (2014). Porous thin membrane barrier layers from 2,3-dicarboxylic acid cellulose nanofibrils for membrane structures. Carbohydr. Ploym..

[14] Visanko M., Liimatainen H., Sirviö J.A., Hormi O. (2015). A cross-linked 2,3-dicarboxylic acid cellulose nanofibril network: A nanoporous thin-membrane layer with tailored pore size for composite membranes. Sep. Purif. Technol..

[15] Wang R., Guan S., Sato A., Wang X., Wang Z., Yang R., Hsiao B.S., Chu B. (2013). Nanofibrous microfiltration membranes capable of removing bacteria, viruses and heavy metal ions. J. Membr. Sci..

[16] Rivera-Utrilla J., Sánchez-Polo M., Gómez-Serrano V., Álvarez P.M., Alvim-Ferraz M.C.M., Dias J.M. (2011). Activated carbon modifications to enhance its water treatment applications. An overview. J. Hazard. Mater..

[17] Busscher H.J., Dijkstra R.J., Engels E., Langworthy D.E., Collias D.I., Bjorkquist W.D., Mitchell M.D., Van der Mei H.C. (2006). Removal of Two Waterborne Pathogenic Bacterial Strains by Activated Carbon Particles Prior to and after Charge Modification. Environ. Sci. Technol..

[18] Ujang Z., Au Y.L., Nagaoka H. (2002). Comparative study on microbial removal in immersed membrane filtration (IMF) with and without powdered activated carbon (PAC). Water Sci. Technol..

[19] Dolić M.B., Rajaković-Ognjanović V.N., Štrbac S.B., Dimitrijević S.I., Mitrić M.N., Onjia A.E., Rajaković L.V. (2017). Natural sorbents modified by divalent Cu^2+^ and Zn^2+^ ions and their corresponding antimicrobial activity. New Biotechnol..

[20] Eltugral N., Simsir H., Karagoz S.B. (2016). Preparation of nano-silver-supported activated carbon using different ligands. Res. Chem. Intermed..

[21] Prashantha Kumar T.K.M., Mandlimath T.R., Sangeetha P., Sakthivel P., Revathi S.K., Ashok Kumar S.K., Sahoo S.K. (2015). Highly efficient performance of activated carbon impregnated with Ag, ZnO and Ag/ZnO nanoparticles as antimicrobial materials. RSC Adv..

[22] Hassan M.L., Bras J., Hassan E.A., Silard C., Mauret E. (2014). Enzyme-assisted isolation of cellulose nanofibers from date palm fruit stalks. Ind. Crops Prod..

[23] Wise L.E., Murphy M., D’Addieco A.A. (1946). Chlorite holocellulose, its fractionation and bearing on summative wood analysis and on studies on hemicelluloses. Pap. Trade.

[24] Browning B.L. (1967). Methods of Wood Chemistry, Vol. 2.

[25] Hassan M.L., Mathew A.P., Hassan E.A., El-Wakil N.A., Oksman K. (2012). Nanofibers from bagasse and rice straw: Process optimization and properties. Wood Sci. Technol..

[26] Saito T., Kimura S., Nishiyama Y., Isogai A. (2007). Cellulose nanofibers prepared by TEMPO-mediated oxidation of native cellulose. Biomacromolecules.

[27] Rwayha Y.M.N., Hassan M.L., Shehata M.R. (2017). Nanoporous activated carbon from olive stones wastes. J. Sci. Ind. Res..

[28] Mautner A., Lee K.-Y., Tammelin T., Mathew A.P., Nedoma A.J., Li K., Bismarck A. (2015). Cellulose nanopapers as tight aqueous ultra-filtration membranes. React. Funct. Ploym..

[29] Gibbins E., Antonio M.D., Nair D., White L.S., Freitas dos Santos L.M., Vankelecom I.F.J., Livingston A.G. (2002). Observations on solvent flux and solute rejection across solvent resistant nanofiltration membranes. Desalination.

[30] Cheng W., He J., Chen M., Li D., Li H., Chen L., Cao Y., Wang J., Huang Y. (2016). Preparation, functional characterization and hemostatic mechanism discussion for oxidized microcrystalline cellulose and its composites. Fiber Ploym..

[31] Wu Y., He J., Cheng W., Gu H., Guo Z., Gao S., Huang Y. (2012). Oxidized regenerated cellulose-based hemostat with microscopically gradient structure. Carbohydr. Ploym..

[32] Dineen P. (1976). Antibacterial activity of oxidized regenerated cellulose. Surg. Gynecol. Obstet..

